# New Cricetid Rodents from Strata near the Eocene-Oligocene Boundary in Erden Obo Section (Nei Mongol, China)

**DOI:** 10.1371/journal.pone.0156233

**Published:** 2016-05-26

**Authors:** Qian Li, Jin Meng, Yuanqing Wang

**Affiliations:** 1 Key Laboratory of Vertebrate Evolution and Human Origins of Chinese Academy of Sciences, Institute of Vertebrate Paleontology and Paleoanthropology, Chinese Academy of Sciences, Beijing, China; 2 Division of Paleontology, American Museum of Natural History, New York, New York, United States of America; Northern Illinois University, UNITED STATES

## Abstract

New cricetids (*Eucricetodon wangae* sp. nov., *Eucricetodon* sp. and *Pappocricetodon siziwangqiensis* sp. nov.) are reported from the lower and middle parts of the “Upper Red” beds of the Erden Obo section in Nei Mongol, China. *Eucricetodon wangae* is more primitive than other known species of the genus from lower Oligocene of Asia and Europe in having a single anterocone on M1, a single connection between the protocone and the paracone, the anterior metalophule connection in M1-2 and weaker anteroconid and ectomesolophid in lower molars. *Pappocricetodon siziwangqiensis* is more advanced than other species of the genus in permanently missing P4 and having posterior protolophule connection. These fossils suggest that the age of the “Upper Red” of the Erden Obo section is younger than the age of the Upper Eocene Houldjin and Caijiachong formations, but older than those containing the Shandgolian faunas; the “Upper Red” is most closely correlative to the Ergilian beds in age, and probably close to the Eocene/Oligocene boundary. Given the age estimate, *Eucricetodon wangae* provides the new evidence to support that cricetid dispersal from Asia to Europe occurred prior to the Eocene-Oligocene boundary.

## Introduction

Cricetids belong to the Muroidea and constitute one of the most diverse families of Rodentia. The oldest cricetids described, *Palasiomys* [1] and *Pappocricetodon* [2], are from middle Eocene sites of China and Kazakhstan [3–5]. Recently many new cricetids have been discovered from the Eocene and Oligocene continental deposits of Asia, such as *Eocricetodon* and *Oxynocricetodon* [6] from late Eocene of Nei Mongol, *Ulaancricetodon* [7] from early Oligocene of Mongolia, *Eucricetodon* [8,9], *Bagacricetodon*, *Plesiodipus*, and *Witenia* [8] from Oligocene of Nei Mongol, *Paracricetops* [10] from early Oligocene of Yunna. These discoveries have provided an important evidence for understanding the origin and early radiation of this family. However, late Eocene cricetids in Asia are poorly known compared to their records in the Oligocene.

Here we report new materials of cricetids from the “Upper Red” of Erden Obo, Nomogen, Siziwangqi, Nei Mongol. These specimens were collected via surface prospecting and screenwashing and represent the first cricetids ever found in this locality and a rare record from the late Eocene of central Asia in general. The discovery not only expands the distribution of the late Eocene cricetids in Asia and increases the diversity of rodents to the local Eocene-Oligocene faunas, but also plays an important role in determining the ages of the fossiliferous beds in these localities and helps for stratigraphic correlation of the Eocene-Oligocene strata in central Asia.

## Material and Methods

**Fig 1:**
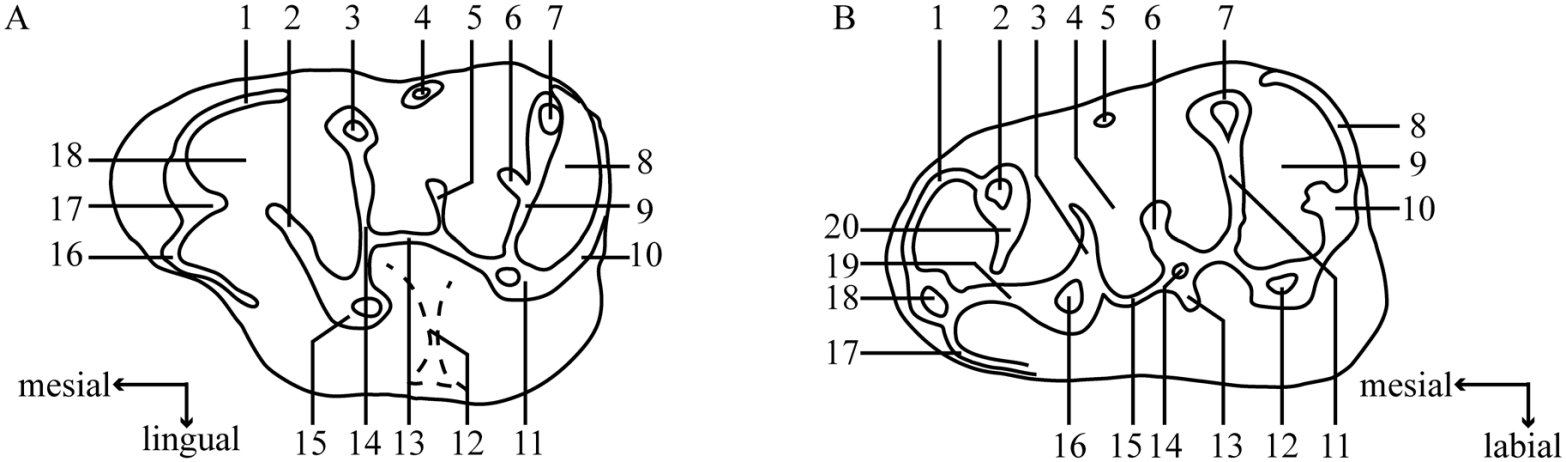
Terminology used in this paper to described molars, modified from Maridet et al. [11]. A, upper molar: 1. labial anteroloph; 2. protocone spur; 3. paracone; 4. mesostyle; 5. mesoloph; 6. neomesoloph; 7. metacone; 8. posterosinus; 9. metalophule; 10. posteroloph; 11. hypocone; 12. sinus; 13. entoloph; 14. protolophule; 15. protocone; 16. lingual anteroloph; 17. anterocone posterior spur; 18. anterocone. B, lower molar: 1. lingual anterolophid; 2. metaconid; 3. protoconid posterior arm; 4. mesosinusid; 5. mesostylid; 6. mesolophid; 7. entoconid; 8. posterolophid; 9. posterosinusid; 10. hypoconid posterior arm; 11. hypolophid; 12. hypoconid; 13. ectomesolophid; 14. mesoconid; 15. ectolophid; 16. protoconid; 17. labial anterolophid; 18. anteroconid; 19. anterolophulid; 20. metalophulid.

All necessary permits were obtained for the described study, which complied with all relevant regulations. Field work in the Erlian Basin were granted by the Department of Land and Resources of Nei Mongol Autonomous Region. All newly described specimens (listed below) were collected in several field expeditions during 2007–2012 by a team from IVPP, American Museum of Natural History, Carnegie Museum of Natural History and Northern Illinois University. All fossil specimens collected belong to and are housed in the Institute of Vertebrate Paleontology and Paleoanthropology (IVPP), Chinese Academy of Sciences in Beijing, and are available for examination by qualified researchers. Dental terminology in the description illustrated in Fig 1, is modified from Maridet et al. [11]. Measurements of teeth were taken using a reticle with an accuracy of 0.1mm mounted in an Olympus SZX7 microscope. SEM photographs of coated teeth were taken using a JSM-6100 SEM machine at the Key Laboratory of Vertebrate Evolution and Human Origins of Chinese Academy of Sciences, Institute of Vertebrate Paleontology and Paleoanthropology, Chinese Academy of Sciences.

### Geological settings

The cricetid specimens that we report here came from the “Upper Red” beds of Erden Obo (Urtyn Obo), Nomogen Sumu, Siziwangqi, Nei Mongol, China. Osborn [12] first reported the Erden Obo section based on Granger’s and Spock’s fieldnotes. He subdivided the deposits in the section into 8 units, termed in descending order as the “Upper White”, the “Upper Red”, the “Middle White or Gray”, the “Middle Red”, the “Lower White”, the “Lower Red”, the “Basal White”, and the “Basal Red”. These units were later referred to as the “Baron Sog Formation”, the “Ulan Gochu Formation”, the “Shara Murun Formation” and the “Arshanto? Formation” [12]. However, the lithological division and correlation of the beds proposed by Osborn [12] have long been a matter of uncertainty and different names of formations and stratigraphic divisions were adopted during last decades [13–17]. Because the complicate research history and potential sedimentary hiatuses in the sequence, the formal division and correlation, including naming of the stratigraphic units, are yet to be completed [17]. For the present, we continue to use the descriptive term “Upper Red” of Osborn to denote the beds where the fossils reported here came from. However, it should be noted that the lithological assignment and age estimate are probably different from those suggested by Osborn.

**Fig 2:**
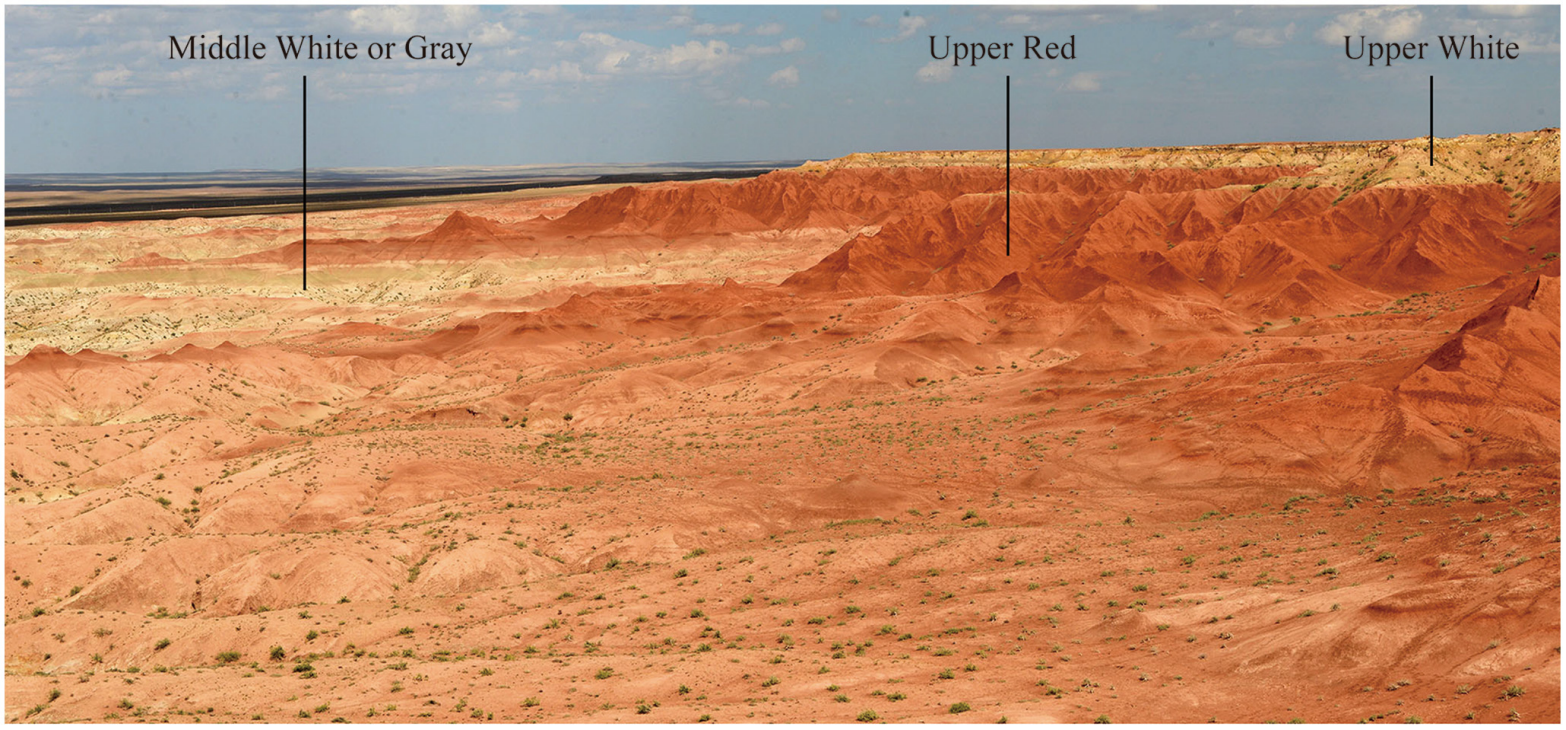
Photograph of the Erden Obo section showing the stratigraphic exposure documenting from the “Middle White or Gray” to the “Upper White”.

All cricetid specimens reported here were collected from the lower and middle part of the “Upper Red” beds (Fig 2); any fossils are rare in the upper part of the “Upper Red” beds.

### Nomenclatural Actas

The electronic edition of this article conforms to the requirements of the amended International Code of Zoological Nomenclature, and hence the new names contained herein are available under that Code from the electronic edition of this article. This published work and the nomenclatural acts it contains have been registered in ZooBank, the online registration system for the ICZN. The ZooBank LSIDs (Life Science Identifiers) can be resolved and the associated information viewed through any standard web browser by appending the LSID to the prefix “http://zoobank.org/”. The LSID for this publication is: urn:lsid:zoobank.org:pub:D422F4AF-A9D3-4C9C-9F78-36FFAF0F4091. The electronic edition of this work was published in a journal with an ISSN, and has been archived and is available from the following digital repositories: PubMed Central, LOCKSS, IVPP-IR.

## Results

### Systematic Paleontology

Class Mammalia Linneaus, 1785

Order Rodentia Bowdich, 1821

Superfamily Muroidea Illiger, 1811

Family Cricetidae Fischer von Waldheim, 1821

Subfamily Eucricetodontinae Mein et Freudenthal, 1971

Genus *Eucricetodon* Thaler, 1966

*Eucricetodon wangae* sp. nov.

urn:1sid:zoobank.org:act:9F09907C-0561-456A-A52A-EB4EA674EF6D

#### Holotype

IVPP V 17807, partial skull with left M1-2 and right M1-3, left mandible with m1 and right mandible with m1-3 from a single individual organism.

#### Referred specimens

IVPP V 17808.1–10, M1; V 17808.11–21, M2; V 17808.22–27, M3; V 17808.28–39, m1; V 17808.40–49, m2; V 17808.50–58, m3.

#### Locality and Horizon

Erden Obo, Nomogen, Siziwangqi, Nei Mongol; lower and middle part of the “Upper Red” beds.

#### Diagnosis

A medium size cricetid. Differs from other known species of *Eucricetodon* in having a single anterocone in M1 (not divided or having a well-developed anterocone posterior spur), a single connection between the protocone and the paracone, an anterior metalophule connection in M1-2, weaker anteroconid and ectomesolophid in lower molars, and opposite position of the two lingual main cusps relative to the two labial ones in lower molars.

#### Etymology

In honor of Banyue Wang, who has made a great contribution to the study of Paleogene cricetids of China.

### Description

#### Skull

**Fig 3:**
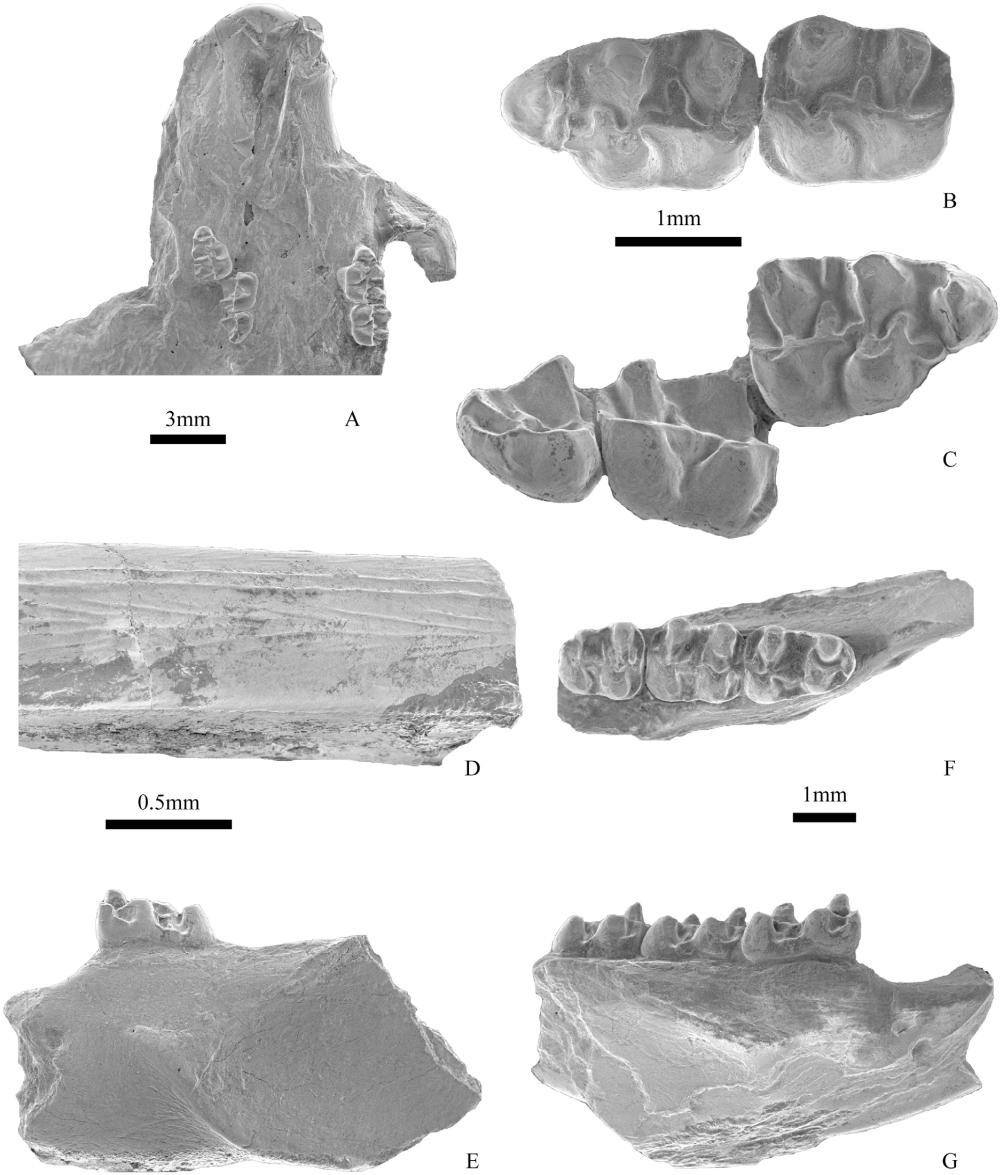
Partial skull and mandibles of *Eucricetodon wangae* sp. nov. (IVPP V 17807, holotype). A, V 17807, ventral view of the skull; B-C, V 17807, left M1-2 and right M1-3 (with the maxillary bone photographically removed); D, V 17807, lower incisor in the right mandible; E, V 17807, labial view of the left mandible; F, V 17807, occlusal view of the right mandible; G, V 17807, labial view of the right mandible. B-C and E-G each to common scale.

The skull is broken and the infraorbital foramen is not preserved in V 17807. The anterior plate on the anteroventral surface of the zygoma is distinct, rounded anteriorly and concave upward. The dorsal ramus of the zygoma is narrow, with greatest thickness in a dorsal-ventral orientation (Fig 3A). The masseteric tubercle forms a rounded prominence on the anteromedial margin of the anterior plate. The incisive foramen is long and oval, and posteriorly positioned on the palate. Because of the distortion, the contributions of the premaxilla and maxilla to the foramen are unclear. The anterior margin of the foramen is 1.5mm posterior to the posterior border of the incisor alveolus, and the posterior margin levels with the anterior edge of the alveolus of M1.

#### Mandible

The diastema is shorter than that of the upper jaw. The masseteric fossa is broad and clearly delimited and the ventral masseteric crest is more distinct than the dorsal one. The anterior edge of the masseteric fossa reaches to the level of the midpoint of m1 (Fig 3E). There are two mental foramina on the lateral surface of the right jaw, which are slightly below the anterior termination of the masseteric crest (Fig 3G).

#### Incisors

The enamel cap covers the labial surface of the lower incisor. Two longitudinal, parallel ridges or raised fine lines are present along the lingual side of the anterior face of the lower incisor, whereas faint lines oblique to the longitudinal ridges are present on the rest part of the anterior face (Fig 3D). The enamel of upper incisors is smoother than that of lower incisor, without ornamentation.

#### Upper Molars

**Fig 4:**
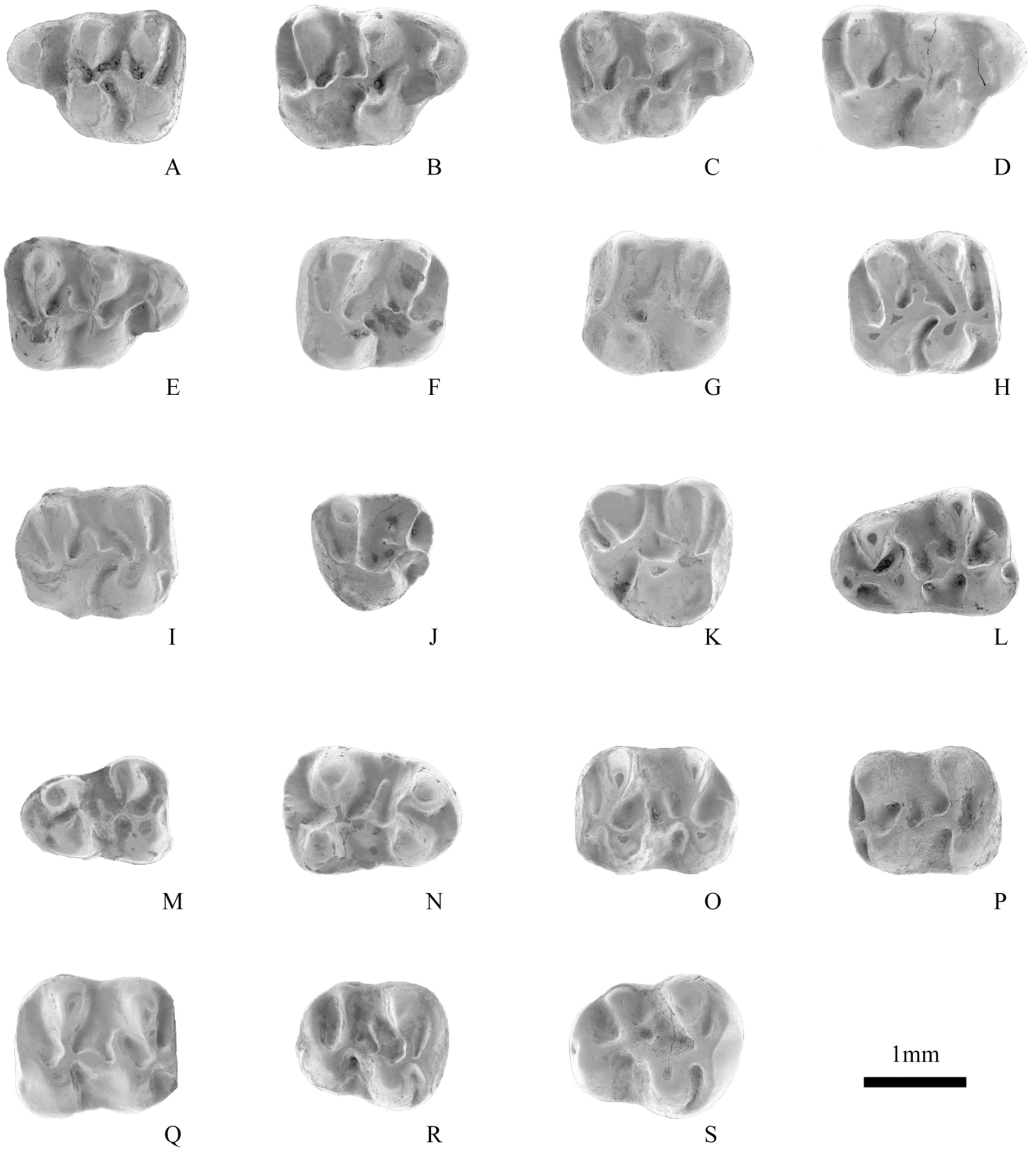
Cheek teeth of *Eucricetodon wangae* sp. nov. in occlusal view. A, IVPP V 17808.3, left M1; B-E, V 17808.7–10, right M1; F-G, V 17808.11, V 17808.13, left M2; H-I, V 17808.17, V 17808.21, right M2; J, V 17808.22, left M3; K, V 17808.26, right M3; L-M, V 17808.28, V 17808.35, left m1; N, V 17808.39, right m1; O-P, V 17808.42–43, left m2; Q, V 17808.48, right m2; R-S, V 17808.54–55, right m3.

M1 bears a strong anterocone located labial to the longitudinal axis of the tooth. The anterocone, transversely elongated and single lobed, has a weak labial anteroloph reaching the paracone and a long lingual anteroloph reaching the protocone on its lower part. The lingual anteroloph swells at its posterior extremity to form a small protostyle in right M1 of V 17807 (Fig 3C). The protocone is slightly oblique, extending posterolingually. The protocone spur (protolophule I) is usually free and short, and ends in the anterosinus (present in 8 specimens of a total of 10 specimens) (Figs 3C, 4A, 4B and 4D), rarely reaches the anterocone (2 of 10) (Fig 4C and 4E). The protolophule (protolophule II) is complete and connects the paracone and the posterior side of the protocone. The mesoloph is moderately elongated. The mesostyle presents in V 17807 (Fig 3C), as the minute spur starting lingually. The metalophule is directed to the anterior part of the hypocone. The entoloph is long and extends obliquely, meeting the protolophule. The posteroloph is thin and long, runs from the hypocone to the posterolabial corner of the tooth to delimit a large posterosinus.

The occlusal surface of M2 is rectangular, being slightly longer than wide (Fig 4F; Table 1). Its anterior border is usually straight, whereas the posterior one is more rounded. The anteroloph has long labial and short lingual arms. A short anterolophule connects the anteroloph to the anterior side of the protocone. The protocone is oblique, more than in M1, and even oriented posteriorly in some cases, so that a narrow sinus between the protocone and the entoloph is delimited (Fig 4H). The protolophule is transverse, and extends from the anterior part of the protocone to the paracone. A mesoloph is always present and the mesocone is present in some case. The entoloph turns lingually at its anterior end to unite with the posterolabial side of the protocone. The lingual extension of the metacone meets the anterior side of the hypocone to complete the metalophule. The thin posteroloph is long, delimiting a large posterosinus as in M1.

**Table 1:** Measurements of teeth of *Eucricetodon wangae* sp. nov. (mm).

Tooth	n	Length		Width	
		Min-max	Mean	Min-max	mean
M1	12	1.75–2.20	1.97	1.30–1.50	1.38
M2	14	1.40–1.70	1.57	1.30–1.65	1.46
M3	07	1.20–1.50	1.31	1.10–1.45	1.26
m1	14	1.40–1.85	1.64	1.00–1.25	1.15
m2	11	1.50–1.80	1.57	1.15–1.40	1.28
m3	10	1.30–1.70	1.53	1.20–1.40	1.33

The M3 has a rounded triangular outline in occlusal view, and is broader anteriorly than posteriorly. It is smaller than M1 and M2. The paracone is the highest cusp on M3. The metacone and the hypocone are reduced, with the metacone being smaller than the hypocone. The protolophule is complete. The metalophule is shorter than the protolophule. The anteroloph and posteroloph are low, and tend to unite with the paracone and metacone, enclosing shallow, narrow anterosinus and posterosinus, respectively. The middle part of the tooth shows a high degree of variation. A neomesoloph branches from the metalophule and extends into the mesosinus, and it varies from being incomplete or long (Fig 4J and 4K). The mesoloph and mesostyle are absent.

The m1 is trapezoidal in occlusal view, and is longer than wide. The anteroconid is distinct, but smaller and lower than any of the main cuspids. The protoconid is usually possesses a front arm, sometimes the front arm forms a short anterolophulid and linked the anteroconid and the protoconid (Figs 3F and 4L). The protoconid and hypoconid are lower than the metaconid and entoconid, respectively. The protoconid posterior arm (metalophulid I) is complete and connected to the metaconid. The hypolophid is complete and extends to the hypoconid or the anterior arm of the hypoconid (Fig 4L, 4M and 4N). The ectolophid is straight and low, connected to the protoconid at its base. A mesoconid is usually present on the ectolophid. The mesolophid is short, but in a few cases it is long (Fig 4N). A small mesostylid exists in some rare cases (Fig 3F). The posterolophid is complete and delimits a large posterosinusid. A short hypoconid posterior arm presents in some teeth (5 of 14) and merges into the posterolophid (Figs 3F and 4L).

The m2 is rectangular in occlusal view and has four main cusps. The anteroconid is absent. Both anterolophids are well developed, but the labial one is short. The metalophulid is long and extends anterolabially to contact the anterolophulid. The protoconid posterior arm is prominent, and either meets the metaconid to close the trigonid basin (8 of 11) (Figs 3F and 4P) or extends almost to the base of the metaconid (3 of 11) (Fig 4Q). The hypolophid is complete and is connected to the ectolophid, anterior to the hypoconid. The ectolophid is usually oblique. The mesolophid is short and the mesoconid is weaker than that in M1. The posterolophid is long and delimiting a broad posterosinusid, but does not always reach the posterolingual side of the entoconid. In some cases the small mesostylid is present. In V 17807 a short hypoconid posterior arm is present (Fig 3F).

The trigonid of m3 is similar to that of m2. A narrow and rounded distal end of m3 is present, and the entoconid is reduced than that in m2. The anterolophids are present, the lingual one is usually longer. The metalophulid bends anteriorly and joins the anterolophid, in some cases it extends further anteriorly at the anterolophulid-anterolophid junction (Fig 4S). The protoconid posterior arm is long, and extends to the mesosinusid in most specimens (5 of 9) (Figs 3F, 4R and 4S), but it joins the base of the metaconid in others (4 of 9). The ectolophid is curved. The hypolophid is complete. The mesoconid and mesolophid are absent.

### Comparison with Asian Eocene and Early Oligocene cricetids

*Eucricetodon wangae* sp. nov. combines some primitive characters (single anterocone in M1, metalophule joining to the anterior part of the hypocone in M1-2) with advanced characters (short protocone spur in M1, posterior protolophule connection in M1, short anterolophulid in m1, long protoconid posterior arm and short hypoconid posterior arm in m1-2). It differs from *Pappocricetodon* [1–6,18,19] in absence of P4 or DP4, having a short protocone spur in M1 and never extending to the anterocone. In addition, its protolophule joins the posterior side of the protocone in M1, the anterolophulid in m1 and hypoconid posterior arm in m1-2 are short. *Eucricetodon wangae* sp. nov. is clearly more advanced than *Pappocricetodon* based on the tooth structures.

*Eocricetodon* and *Oxynocricetodon* were first described by Wang and Meng [20], including four species, *Eo*. *meridionalis*, *Eo*. *borealis*, *O*. *leptaleos* and *O*. *erenensis* from the late Eocene of the Caijiachong Formation of Qujin of Yunna, and the Houldjin Formation of Nei Mongol [6]. *Eucricetodon wangae* sp. nov. is larger than *Eocricetodon* (Table 2). It differs from *Eocricetodon* in having a short protocone spur in M1, a short m1 anterolophulid that links the anteroconid and the protoconid, and a short hypoconid posterior arm in m1-2. In contrast, *Eocricetodon* usually has a long protocone spur that extends to the anterocone, occasionally presents a short protocone spur in *Eo*. *meridionalis*. *Eo*. *borealis* has a distinct protoconule on long protolophule. In *Eocricetodon* m1 anterolophulid and hypoconid posterior arm on m1-2 are absent.

**Table 2:** Comparison of measurements among *Eucricetodon*, *Eocricetodon* and *Oxynocricetodon* (mm).

	*Eucricetodon wangae* sp. nov.	*Eucricetodon asiaticus*	*Eucricetodon caducus*	*Eucricetodon deploratus*	*Eucricetodon* aff. *E*.*caducus*	*Eucricetodon borealis*	*Eucricetodon meridionalis*	*Oxynocricetodon erenesis*	*Oxynocricetodon leptaleos*
M1(L)	1.75–2.20	2.02–2.44	2.17	2.43	1.85–2.25	1.60	1.65–1.84	2.05	1.53–1.65
M1(W)	1.30–1.50	1.34–1.50	1.48		1.28–1.53	1.04–1.15	1.00–1.31	1.46	1.00–1.06
M2(L)	1.40–1.70	1.47–1.66	1.32	1.90	1.80–1.67	1.30–1.34	1.47–1.50	1.60	1.19–1.28
M2(W)	1.30–1.65	1.43–1.62	1.32		1.21–1.51	1.18–1.30	1.18–1.34	1.50	1.09–1.19
M3(L)	1.20–1.50	1.18–1.40			1.14–1.37	0.90	1.28–1.34	1.35–1.50	0.97–1.13
M3(W)	1.10–1.45	1.12–1.37			1.11–1.28	1.00	1.23–1.28	1.20	0.91–1.05
m1(L)	1.40–1.85	1.60–2.16	1.58		1.44–1.90	1.30–1.75	1.38–1.53		1.16–1.36
m1(W)	1.00–1.25	1.10–1.40	1.20		0.96–1.37	0.95–1.36	0.97–1.06		0.91–1.03
m2(L)	1.50–1.80	1.40–1.86	1.34–1.57		1.09–1.72	1.50–1.60	1.38–1.50	1.60	1.28–1.31
m2(W)	1.15–1.40	1.06–1.52	1.10–1.29		1.07–1.39	1.20–1.25	1.13–1.25	1.35–1.36	1.06–1.09
m3(L)	1.30–1.70	1.32–1.73			1.32–1.61		1.41	1.70	1.16–1.25
m3(W)	1.20–1.40	0.96–1.40			1.08–1.33		1.91–1.25	1.30	0.91–1.06

Note for table 2: Data for *Eucricetodon asiaticus* from Lindsay [21]; for *Eucricetodon caducus* from Wang [9]; for *Eucricetodon deploratus* from Shevyreva [22]; for *Eucricetodon* aff. *E*. *caducus* from Maridet et al. [11]; for *Eocricetodon borealis* and *Oxynocricetodon erenensis* from Wang [6]; for *Eocricetodon meridionalis* and *Oxynocricetodon leptaleos* from Wang and Meng [20].

*Eucricetodon wangae* sp. nov. is similar to those of *Oxynocricetodon* in having a short protocone spur that never merges with the anterocone, but their differences are distinct. Upper molars of *Oxynocricetodon* have anteriorly jointed protolophule and metalophule, but the protolophule M1 of *Eucricetodon wangae* sp. nov. is short and joins the protocone on its posterior side. However, the anteriorly jointed protolophule in M1 indicates that *Oxynocricetodon* is more primitive than *Eucricetodon wangae* sp. nov.. The M2 entoloph of *O*. *erenensis* is long and meet both the protocone spur and the protolophule to close a pit, contrasting a shorter entoloph in *Eucricetodon wangae* sp. nov. that joins the posterolabial side of the protocone. In the lower teeth of *Eucricetodon wangae* sp. nov., the protoconid posterior arm of m2 is longer and usually closes to the trigonid basin, while the hypoconid posterior arm on m1-2 is short in some cases.

In the early Oligocene some species of *Eucricetodon*, *Cricetops* and *Selenomys* were widely distributed all over Asia. In Mongolia they are known from different areas [23,24] and coexist with *Ulaancricetodon* in the Valley of Lakes [7]. Recently, new materials of cricetids from the Ulantatal area of Nei Mongol are reported [8], including *Eucricetodon*, *Bagacricetodon*, *Plesiodipus*, *Cricetops*, *Pseudocricetops*, *Witenia*. *Eucricetodon wangae* sp. nov. shows distinct differences from *Selenomys*, *Cricetops*, *Ulaancricetodon*, *Bagacricetodon*, *Witenia*, and *Pseudocricetops*. Among these cricetids, *Cricetops* and *Psuedocricetops* have two anterocones in M1, and some cases of *Bagacricetodon* have divided anterocone. *Witenia* is a cricetid with large size; it has double or incipiently double protolophule in M2, deeper sinus in M3, and has more-developed ectolophid in lower molars. *Bagacricetodon* lacks the posteroloph and has strong retroverse lophs in upper molars, the metalophulid reaching the anteroconid in m1, short or absent protoconid posterior arm in m2. The anterior lobe in M1 of *Ulaancricetodon* is small, without anterocone. Unlike other Oligocene cricetids, *Ulaancricetodon* has long and strong oblique protocone spur that extends to the antero-buccal edge, double protolophule, and well-developed mesoloph and mesolophid.

The new specimens from the “Upper Red” of the Erden Obo section possess many common characters in *Eucricetodon* such as bunodont teeth, the simple lophs, a single anterocone (anteroconid) in M1 (m1), and present a short hypoconid posterior arm. However, the new species is distinctive from other known species of the Oligocene *Eucricetodon*. There are five species of *Eucricetodon* from Oligocene of Asian known to date: *E*. *asiaticus*, *E*. *caducus*, *E*. *deploratus*, *E*. *jilantaiensis*, *E*. *bagus* and *E*. aff. *E*. *caducus*.

*E*. *asiaticus* was established by Matthew and Granger [24] and described in detail by Lindsay [21] based on materials from the Hsanda Gol Formation, Mongolia. Some specimens of *E*. *asiaticus* also were reported from the Ulantatal area of Nei Mongol [8]. *Eucricetodon wangae* sp. nov. differs from *E*. *asiaticus* in several aspects of the cheek teeth, including the anterior connection of the metalophule in M1-2 (posterior connection in the type of *E*. *asiaticus*), a single protolophule connection between the paracone and the protocone (the connection is double in some cases of *E*. *asiaticus*), more labial anterocone and absent short anterocone posterior spur in M1, more smaller anteroconid and weaker anterolophulid in m1, and the rareness of the ectomesolophid.

*E*. *caducus* and *E*. *deploratus* were from the early Oligocene of Kazakhstan [22] and they are larger than *Eucricetodon wangae* sp. nov. (Table 2). Wang [9] proposed an emended diagnosis of *E*. *caducus* based on the early Oligocene material from the Nei Mongol. *Eucricetodon wangae* sp. nov. is similar to *E*. *caducus* in several aspects, such as the anterior connection of the protolophule in M2 and the metalophule in M1-2, a single protolophule in M2, a small anteroconid and a complete protoconid posterior arm in m1. However, *Eucricetodon wangae* sp. nov. differs from *E*. *caducus* in lacking a close protosinus between the protocone and the paracone, having the entoloph connecting with the posteriolabial side of the protocone in M2, and a long protoconid posterior arm in m2. *E*. *deploratus* differs from *Eucricetodon wangae* sp. nov. in having more massive tooth cusps, an entoloph more lingually located, the lingual anteroloph of M2 is weakly developed.

*E*. *jilantaiensis* and *E*. *bagus* were described from the early late Oligocene of the Ulantatal area of Nei Mongol [8]. They show more differences from *Eucricetodon wangae* sp. nov.. *E*. *jilantaiensis* has posterior connection of the protolophule and metalophule in M1-2, is absent protocone spur in M1, has well-developed metalophulid in m1. The double connection between the anterocone and protocone is present in M1 and M2 of *E*. *bagus*. Similar to *E*. *jilantaiensis*, the protolophule and metalophule of *E*. *bagus* are posterior connection. In the lower teeth of *E*. *bagus*, the anteroconid in m1 is isolated, the protoconid posterior arm is short and the mesolophid is absent in m2.

Maridet et al [11] described *E*. aff. *E*. *caducus* from the late Oligocene Tieersihabahe Formation of the Junggar basin, northern Xinjiang. It differs from the *Eucricetodon wangae* sp. nov. in having a slight bilobed anterocone of M1 in some case, a weak-developed second mesoloph and mesolophid in some specimens, present ectomesolophid in m1, more weaker mesoconid in m1, more shorter protoconid posterior arm and not closing the trigonid basin in m2.

### Comparison with European Oligocene *Eucricetodon*

Cricetids appeared in Europe after the Grande Coupure, where they dispersed and diversified rapidly [10,11,25,26]. *Atavocricetodon* from Hoogbutsel (Belgium) is the oldest cricetid in the European record (early Oligocene, MP21). *Atavocricetodon* includes *A*. *atavus* [27], *A*. *atavoides*, *A*. *hugueneyae*, *A*. *nanus* and *A*. *minusculus* [28], all known from the early Oligocene of Europe. De Bruijn et al. [29] proposed that *Atavocricetodon* may be a “morpho-subgenus” containing the oldest forms of *Eucricetodon* known in the early Oligocene of Europe. Based on the morphometrical and microstructural analyses, Gomes Rodrigues et al. [30] pointed that *Atavocricetodon* cannot be retained either as a genus, or as a morphosubgenus of *Eucricetodon*. Maridet and Ni [10] suggested that *Atavocricetodon* is the sister taxon of *Eucricetodon* in their phylogenetic analysis. The relationship of *Atavocricetodon* and *Eucricetodon* is not the key point of this paper and *Atavocricetodon* is still regarded as one of the primitive cricetids of Europe. *Eucricetodon wangae* sp. nov. is similar to *Atavocricetodon* in many dental characters, such as the position of the anterocone, the posterior connection of the protolophule in M1, the anterior connection of the metalophule in M1-2, the incomplete protocone spur, the oblique shape of the protocone in M1-2. However, *Atavocricetodon* shows some differences with *Eucricetodon wangae* sp. nov., including presence of the anterocone posterior spur in M1, commonly complete anterolophulid, a well-developed metalophule, usually present ectomesolophid and hypoconid posterior arm in M1 of *A*. *atavoides* and *A*. *hugueneyae*, absent mesoloph in *A*. *minusculus*.

The genus *Eucricetodon* is more diversified in Europe than in Asia. *E*. *huberi*, *E*. *quercyi*, *E*. *hochheimensis*, *E*. *collatus*, *E*. *haslachensis*, *E*. *gerandianus* [31], *E*. *dubius* [31,32], *E*. *martinensis* [33], *E*. *huerzeleri* [26], *E*. *hesperius*, *E*. *longidens* [25,34] are recorded from the Oligocene Europe. *Eucricetodon wangae* sp. nov. basically differs from all of them by its single anterocone in M1 (never divided or present well-developed anterocone posterior spur), a single connection between the protocone and the paracone, the anterior metalophule connection in M1-2, more weaker anteroconid and ectomesolophid in lower molars and opposite position of the two lingual main cusps to the two buccal cusps in lower molars.

### *Eucricetodon* sp.

#### Specimens

IVPP V 17809.1–4, M1; V 17809.5–7, m1.

#### Locality and Horizon

Erden Obo, Nomogen, Siziwangqi, Nei Mongol; lower part of the “Upper Red” beds.

### Description and comparisons

**Fig 5:**
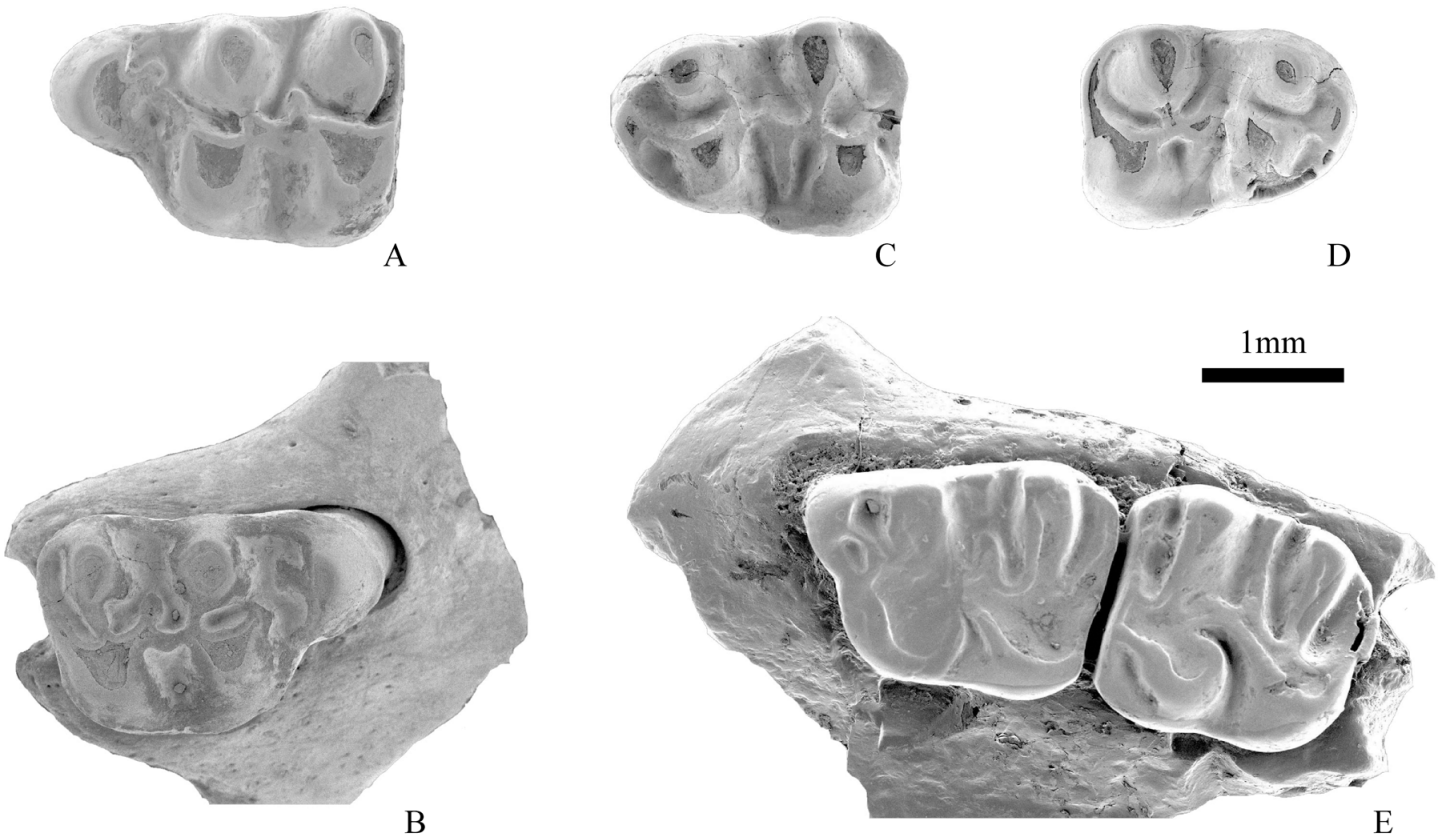
A-D, Cheek teeth of *Eucricetodon* sp. in occlusal view: A, IVPP V 17809.1, left M1; B, V 17809.3, right M1; C, V 17809.4, left m1; D, V 17809.7, right m1; 5E. V 17806, occlusal view of M1-2 of *Pappocricetodon siziwangqiensis* sp. nov.

M1 bears a strong anterocone, develops a weak anterocone posterior spur that extends toward but never reaching to the protocone. The main four cusps are massive and the lophs are short. The protocone spur usually is medium length and merged into the anterosinus (2 of 4) (Fig 5A), but in some cases it is short (2 of 4) (Fig 5B). The posterior protolophule connection between the protocone and the paracone is short. The metalophule is directed to the middle part of the hypocone. The mesoloph is short and the mesocone is absent. The entoloph is complete, but short. The posteroloph is long and thin, reaching the metacone on the posterolabial side.

The anteroconid in m1 is conical, central or slight labial, and smaller and lower than any of the main cuspids. The anteroconid connects to the protoconid by a short anterolophulid (Fig 5C). The anteroconid and the metaconid are isolated from each other. The protoconid posterior arm is complete and connected to the metaconid (Fig 5C and 5D). A short hypolophid extends to the anterior arm of the hypoconid. A distinct mesoconid presents on the straight ectolophid. The mesolophid is short, but the ectomesolophid is well-developed. The posterolophid is complete and delimits a large posterosinusid. The hypoconid posterior arm is absent.

These specimens are similar to *Eucricetodon* in bearing a single anterocone in M1, the globulous cusps or cuspids, and the simple lophs. They differ from the known species of the genus in having a larger size (Table 3), more massive cusps or cuspids, shorter lophs. In the known Oligocene species of *Eucricetodon*, *E*. *deploratus* [22], *E*. *hochheimensis* and *E*. *haslachensis* [31,34] also have more elongated cusps. The lingual cusps of *E*. *deploratus* are more reduced than the labial ones, but the lingual and labial cusps in our specimens are similar in size. The *E*. *hochheimensis* and *E*. *haslachensis* bear larger anterocone, longer mesoloph and a long hypoconid posterior arm in m1-2. Here these teeth are tentatively referred to *Eucricetodon*, but tentatively regarded as indeterminate at the specific level.

**Table 3:** Measurements of teeth of *Eucricetodon* sp. (mm).

	M1	M1	M1	M1	m1	m1	m1
	V17809.1	V17809.2	V17809.3	V17809.4	V17809.5	V17809.6	V17809.7
L	2.50		2.45	2.45	2.10	2.15	1.95
M	1.70	1.65	1.60	1.55	1.55	1.35	1.45

Subfamily Pappocricetodontinae Tong, 1992

Genus *Pappocricetodon* Tong, 1992

*Pappocricetodon siziwangqiensis* sp. nov.

urn:1sid:zoobank.org:act:D945C4CB-EF11-4240-906F-815FEC480A5D

#### Holotype

IVPP V 17806, a left maxilla with M1-2 (Fig 5E).

#### Locality and Horizon

Erden Obo, Nomogen, Siziwangqi, Nei Mongol; lower part of the “Upper Red” beds.

#### Etymology

The specific name is derived from Siziwangqi, the county where the type locality is located.

#### Diagnosis

Differs from other known species of *Pappocricetodon* in having larger size, a posterior connection of the protolophule in M1 and absence of P4.

### Description

Tooth measurements from the holotype (length/width in mm) are: 2.2/1.7 for M1 and 1.9/1.8 for M2. M1 is trapezoidal in occlusal view, with the labial wall being slightly longer than the lingual. Its small anterior lobe has a small anterocone. The protocone is bulbous and positioned slightly more labially than the hypocone. The protocone spur is long and reaches the distal base of the anterocone. The paracone and metacone are transversely wide and subequal. The protolophule joins the paracone on its posterior side. The metalophule is long and joins the hypocone in its middle part. The mesoloph is transverse, from medial in position to closer to the metalophule and midlength. The mesostyle has a distinct lingual crest extending toward the mesoloph. The posteroloph is thin and low, and extends labially to the posterior side of the metacone.

M2 is rectangular in occlusal view. The labial anteroloph is straight and separated from the paracone by a shallow groove. The labial end of the labial anteroloph inflates as a small anterocone. The protocone is more oblique than in M1. The protolophule is complete and joins the anterior part of the protocone. The entoloph is long, its anterior end turning lingually to the labial side of the protocone. The mesocone is not recognizable. The mesoloph is complete and long. The metalophule joins the hypocone at its anterior side. The thin posteroloph is long, delimiting a large posterosinus as in M1.

### Comparisons

*Pappocricetodon siziwangqiensis* sp. nov. differs from late Eocene and early Oligocene Asian cricetids, such as *Eocricetodon*, *Oxynocricetodon* [6,20], *Eucricetodon* [8,9,21,24], *Paracricetops* [10], *Ulaancricetodon* [7], *Witenia* [8,29], *Cricetops* and *Selenomys* [9,24] in several features: the M1 anterocone is a small cusp in *P*. *siziwangqiensis* sp. nov., but is larger in *Eocricetodon*, and becomes more distinct in *Oxynocricetodon*, *Eucricetodon* and *Paracricetops*. The M1 anterocone of *Cricetops* consists of well-developed twin cusps. *Selenomys* molars are characterized by a selenodont-type and semi-hypsodont morphology. *P*. *siziwangqiensis* sp. nov. is similar to *Eocricetodon* in having the M1 protocone spur extended to the anterocone, whereas that of *Oxynocricetodon* and *Eucricetodon* is short, free and separated from the anterocone. The M1 protolophule of *P*. *siziwangqiensis* sp. nov. meets the posterior side of the protocone, but that of *Oxynocricetodon* joins the anterior side of the protocone. The metalophule of *P*. *siziwangqiensis* sp. nov. is an anterior connection, contrasting to a posterior one in some species of *Eucricetodon*. *P*. *siziwangqiensis* sp. nov. is similar to *Ulaancricetodon* in several aspects, such as having a small anterocone in M1, the long protocone spur and extending to the anterolabial edge of M1, the M1 protolophule joining the protocone on its posterior side. However, *P*. *siziwangqiensis* sp. nov. differs from *Ulaancricetodon* in having a shorter mesoloph, lack of the second mesoloph in M1 and having only one protolophule that connects the protocone and the paracone in M2.

*Witenia* [29] was described from the Eocene/Oligocene boundary near Süngülü of Turkey, and some materials from the Ulantatal area were later assigned to *Witenia* [8]. Based on a lophate pattern, a crescentic anteroconid and m1 smaller than m2, *Witenia* was referred to Pappocricetodontinae. However, *Witenia* appears morphologically advanced over *P*. *siziwangqiensis* sp. nov. in having a more developed anterocone and more lingually placed, double or incipiently double metalophule in M2.

*Pappocricetodon* is one of the primitive cricetid genera, which includes five species: *P*. *antiquus* from Liyang, Jiangsu and Erden Obo, Nei Mongol [5,6]; *P*. *schaubi* from the Zhaili Member of the Hedi Formation Yuanqu, Shanxi Province [1,2,18,19]; *P*. *rencunensis* from the Rencun Member of the Hedi Formation Mianchi, Henan Province [2]; *P*. *kazakstanicus* from the Shinzhaly fauna of East Kazakstan [3] and *P*. *neimongolensis* from the Irdin Manha Formation of Huheboerhe area, Nei Mongol [4]. The new specimens from the Erden Obo section possess many characters common in *Pappocricetodon*, such as the brachyodont cheek teeth, a bulbous protocone in M1 and M2, a less enlarged M1 with small anterocone and a long protocone spur extending to the anterocone. These characteristics strong support the attribution of the new specimens to *Pappocricetodon*. *P*. *siziwangqiensis* sp. nov. is also larger than *P*. *antiquus*, *P*. *schaubi*, *P*. *rencunensis*, *P*. *kazakstanicus*, and *P*. *neimongolensis* and differs from those species in having a short protolophule that joins the paracone on its posterior side and absence of P4. A small P4 is present in *P*. *antiquus*. In *P*. *neimongolensis*, *P*. *kazakstanicus*, and *P*. *rencunensis* the anterior surface of M1 in some cases has a small interdental wear facet, indicating that a small P4 was present. There is no alveolar and no interdental wear facet on M1 of *P*. *siziwangqiensis* sp. nov. which suggests that P4 has lost in the new species. In *P*. *neimongolensis*, *P antiquus* and *P*. *kazakstanicus* the protolophule extends to the anterior part of the protocone and the protoconule on protolophule is present. Double connection between the protocone and the paracone occurs in *P*. *rencunensis* and sometimes in *P*. *schaubi*. Absence of P4 and the protolophule joining to the posterior part of the protocone indicate that *P*. *siziwangqiensis* sp. nov. is more advanced than others species of the genus.

## Discussion

*Eucricetodon wangae* sp. nov., *Eucricetodon* sp. and *Pappocricetodon siziwangqiensis* sp. nov. were found in the lower and middle part of the “Upper Red” beds of the Erden Obo section.

*Pappocricetodon siziwangqiensis* is more advanced than other species of *Pappocricetodon* in absence of P4 and having posterior protolophule connection. *Pappocricetodon* is known from late middle through late Eocene [1–6,18]. Based on the evolutionary trends of the early cricetids [1,2,6], *Eucricetodon wangae* is more advanced than *Eocricetodon* and *Oxynocricetodon* from the late Eocene of the Houldjin and Caijiachong Formation because it has a distinct anterocone in M1, a short protocone spur in M1 and free from anterocone, a posterior protolophule connection, a complete entoloph, a long protoconid posterior arm in m1-2, and the short hypoconid posterior arm on m1-2 in some case. In known species of *Eucricetodon*, *Eucricetodon wangae* is most similar to *E*. *caducus* from the early Oligocene of Kazakhstan [22] and *Atavocricetodon* from early Oligocene of Europe [28]. However, *Eucricetodon wangae* is more primitive than other known species of *Eucricetodon* from Oligocene Asian and European (included *Atavocricetodon*) in having a single anterocone in M1 (never divided or present well-developed anterocone posterior spur), a single connection between the protocone and the paracone, the anterior metalophule connection in M1-2, more weaker anteroconid and ectomesolophid in lower molars.

Many paleontologists accepted the Asian continental Eocene-Oligocene boundary correlates closely with Ergilian/Shandgolian [35]. The Ergilian was characterized by the Ergilin Dzo fauna of Mongolian, and the Shandgolian was based on the Hsanda Gol fauna in the Valley of Lakes area in Central Mongolia. The sediment sequences of the Hsanda Gol Formation, long known for their fossil richness and basaltic volcanism, allow a stratigraphic adjustment based on the evolution of mammals and on the age determinations of basalts [7,36]. Based on the actual stratigraphic ranges of mammalian genera in the Ergilian Dzo and Hsanda Gol, Dashzeveg [37] provided the Ergilian and Shandgolian mammal assemblage. The rodent is rarely discovered from the Ergilin Dzo fauna (only *Eucricetodon* ad *Pseudocylindrodon*), but rodents are highly diverse in the Hsanda Gol fauna, including 6 families and 12 genera. Recently, in the frame of a Mongolian-Austrian project, 289 fossil taxa were collected from 85 fossil horizons of 33 fossil sites of the Hsanda Gol and the Loh Formations of the Valley of Lakes [38]. By integrating the new data on large and small mammals, the Mongolian informal biozones A, B, C, C1 were updated. The cricetid fossils representing biozone A are *Eucricetodon caducus*, *Eucricetodon asiaticus*, *Selenomys minicus*, *Cricetops dormitor* and *Ulaancricetodon badamae*.

Emry et al. [39] reported the Zaysan Basin of eastern Kazakstan, and suggested the Kusto-Buran interval is with in the Ergilian-Shandgolian transition. Several mammal assemblages from different levels within this Aksyir-Buran unit have been collected. Only one species of Cricetidae (*Eucricetodon* sp.) occurs in the upper part of the Aksyir Svita, and the mammal assemblage of this stratigraphic unit is clearly Ergilian. Within the upper part of the Kusto-Buran unit, the fauna contains *Cricetops*, *Eucricetodon asiaticus*, *Tataromys*, *Tsaganomys* and other taxa, indicative of Shandgolian age.

Ye et al. [40] investigated the Keziletuogaiyi Formation of the Burqin Basin in the northern area of Xinjiang, and their works implied that these beds in Burqin Basin are lithologically different from but biostratigraphically correlative with the Ergilian-Shandgolian sediments in Mongolia and in Zaysan Basin of Kazakhstan. The assemblage from bed 19 is dominated with perissodactyls and is similar to the Ergilin faunas of Mongolia, but the rodent fossil is absent here. The assemblage from bed 22 is comparable to the early Shandgolian faunas, and is including cricetids *Cricetops dirmitor* and *Eucricetodon* sp. The younger assemblage from bed 28 is comparable to Mongolia biozone A and is dominated with small mammals, such as *Cricetops dormitor*, *Eucricetodon asiaticus*, *E*. *caducus* and *Ulaancricetodon* cf. *U*. *badamae* of cricetids.

Obviously, *Cricetops dormitor* and *Selenomys mimicus*, the typical taxa in the early Shandgolian fauna or Mongolian biozone A, are absent in the cricetid assemblage from the “Upper Red” of the Erden Obo section. *Eucricetodon wangae* is more primitive than *E*. *asiaticus* and *E*. *caducus* that are present in Mongolian biozone A. All considered, the cricetid fossil evidence suggests that the age of the “Upper Red” of the Erden Obo section is older than Shandgolian and similar to the Ergilian. In addition, *Pappocricetodon siziwangqiensis* is more advanced than other species of *Pappocricetodon*, and *Eucricetodon wangae* is more advanced than *Eocricetodon* and *Oxynocricetodon* from the late Eocene of the Houldjin and Caijiachong Formation, so the age of the “Upper Red” is later than the age of the Houldjin and Caijiachong Formation, and may be close to the Eocene/Oligocene boundary.

The stronger affinity of Asian cricetid species with the Oligocene European species is already noted by Lindsay [21] and Maridet et al. [11]. *Eucricetodon wangae* from the “Upper Red” also shows a morphological affinity with European early Oligocene *Atavocricetodon*. Baciu and Hartenberger [41] suggested that *Eucricetodon* and *Pseudocricetodon* have migrated to Eastern Europe in late Eocene. Maridet and Ni [10] deduced that the first diversification and dispersal of the family Cricetidae across Eurasia must have occurred well before the Eocene-Oligocene transition. Discovery of *Eucricetodon wangae* from the late Eocene in Nei Mongol enhances this opinion. These late Eocene Asian migrants may have served as a stock for European faunal reorganization during the “Grande Coupure” and the later rapid diversification of Oligocene European cricetids.

Except the cricetids from the “Upper Red” of the Erden Obo section, the ctenodactyloid, the cylindrodontid and the zapodid were also found in the same beds. The detailed study of those taxa will be carried out in separate studies. Meng and McKenna [35] suggested “the Mongolian Remodelling” and interpreted that the Eocene-Oligocene distinct faunal turnovers (perissodactyls-dominant faunas of the Eocene were abruptly replaced by rodent/lagomorph dominant faunas of Oligocene) is marked by global climate change. To more deeply understand the significance of these faunal turnovers, we need to know more about the mammal faunas of the late Eocene and the earliest Oligocene of China. Obviously, the abundant rodents of the Erden Obo section are much informative to realize the diversification of rodents during the Eocene/Oligocene boundary.
